# Double Parathyroid Carcinoma Associated With CDC73 Mutation: A Rare Case

**DOI:** 10.7759/cureus.95382

**Published:** 2025-10-25

**Authors:** Sandra Baptista, Helena Leandro, Catarina Gama, Dora Lameiras, Ana Alves Rafael, Bernardo Marques, Martinha Chorão, Tiago Saldanha

**Affiliations:** 1 Medical Oncology Department, Lisbon Portuguese Institute of Oncology Francisco Gentil, Lisbon, PRT; 2 General Surgery Department, Hospital Egas Moniz – Lisbon Western Local Health Unit (ULSLO), Lisbon, PRT; 3 Endocrinology Department, Hospital Egas Moniz – Lisbon Western Local Health Unit (ULSLO), Lisbon, PRT; 4 Department of Internal Medicine 4, Hospital Egas Moniz – Lisbon Western Local Health Unit (ULSLO), Lisbon, PRT; 5 Pathological Anatomy Department, Hospital Egas Moniz – Lisbon Western Local Health Unit (ULSLO), Lisbon, PRT; 6 Radiology Department, Hospital Egas Moniz – Lisbon Western Local Health Unit (ULSLO), Lisbon, PRT

**Keywords:** brown tumors, cdc73 mutation, hungry bone syndrome, osteitis fibrosa cystica, parathyroid carcinoma, primary hyperparathyroidism

## Abstract

Primary hyperparathyroidism is a relatively common endocrine disorder, but its malignant form is extremely rare and often presents diagnostic and therapeutic challenges. We present an unusual case involving a man in his late 40s who presented with progressive musculoskeletal pain, functional decline, and weight loss. Laboratory tests revealed severe hypercalcemia and markedly elevated parathyroid hormone levels. Imaging identified multiple osteolytic lesions, and biopsy confirmed osteitis fibrosa cystica. Cervical imaging revealed two suspicious parathyroid nodules, raising concern for malignancy. The patient underwent en bloc resection of the left parathyroid and hemithyroidectomy, followed by right inferior parathyroidectomy and left central compartment lymphadenectomy. Histopathological analysis confirmed parathyroid carcinoma with thyroid invasion on the left and an atypical neoplasm on the right, although capsular invasion could not be assessed. Postoperatively, the patient developed hungry bone syndrome, requiring calcium and vitamin D supplementation. Genetic testing revealed a *CDC73* mutation, confirming the genetic basis of the disease.

## Introduction

Primary hyperparathyroidism (PHPT) is an endocrine disorder caused by excessive secretion of parathyroid hormone (PTH), which, under physiological conditions, maintains calcium and phosphate homeostasis through its actions on bone, kidneys, and the gastrointestinal tract [1,2]. PHPT is often asymptomatic and typically identified incidentally through routine biochemical testing revealing hypercalcemia. Diagnosis is confirmed by elevated PTH levels in the presence of hypercalcemia [1-3]. Cortical bone is predominantly affected by resorption, increasing the risk of fractures. In advanced cases, PHPT may manifest as osteitis fibrosa cystica, characterized by fibrous tissue and multinucleated giant cells replacing normal bone, so-called “brown tumors” [1, 2].The condition is most commonly caused by a solitary benign parathyroid adenoma, accounting for approximately 85% of cases [1]. Less frequently, PHPT results from multiglandular disease, either synchronous or asynchronous adenomas, which represent around 15% of cases [1]. Parathyroid carcinoma (PC) and atypical adenomas are rare, comprising fewer than 1% of cases [1,4]. The synchronous presentation of both entities is exceptionally rare, with only isolated cases reported in the literature, making this case particularly noteworthy. PC typically presents in the fifth decade of life and affects both sexes equally [4]. It may occur sporadically or in association with hereditary syndromes such as multiple endocrine neoplasia type 1 (MEN1) or hyperparathyroidism-jaw tumor syndrome (HPT-JT). Mutations in the *CDC73* gene are strongly implicated in both sporadic and familial forms [5].

## Case presentation

A man in his late 40s, with a 17 pack-year smoking history and no significant past medical history, presented to the Emergency Department with posterior right thigh pain following a fall. He also reported chronic right knee pain for the past three months, which was unresponsive to analgesics, accompanied by progressive loss of ambulation. Over the preceding year, he had lost 15 kg (approximately 15% of body weight), with associated nausea, anorexia, myalgias, polyuria, polydipsia, asthenia, and worsening fatigue. He denied neurological, gastrointestinal, urinary, dermatological, or constitutional symptoms such as fever or night sweats. He was not on regular medication, had no recent drug exposures, and denied supplement use. Family history included a sister with hypercalcemia of unknown cause during pregnancy.

On examination, he was alert, afebrile (36.9°C), hemodynamically stable (blood pressure 129/72 mmHg, heart rate 95 bpm, respiratory rate 18 breaths per minute), well-perfused, and mildly dehydrated. There were no palpable cervical masses. Cardiopulmonary and abdominal examinations were normal. He had right lower limb claudication due to pain, without neurological deficits or inflammatory signs.

Initial laboratory tests revealed hypercalcemia (13.9 mg/dL), hypophosphatemia (1.1 mg/dL), and low 25-hydroxyvitamin D levels (25.8 nmol/L). PTH was markedly elevated (1,012 pg/mL), consistent with PHPT. Renal function and albumin levels were normal; alkaline phosphatase was elevated (240 U/L), indicating increased bone turnover. Hypercalciuria (429 mg/24h) excluded familial hypocalciuric hypercalcemia. Multiple myeloma was ruled out by the absence of monoclonal proteins and a negative Bence-Jones protein test. Other laboratory results, including complete blood count, thyroid function, were unremarkable (Table 1).

**Table 1:** Laboratory investigations and results. Bold values indicate findings consistent with primary hyperparathyroidism.

	Results	Interpretation
Hematology		
Hemoglobin	13.4	13.0-17.0 g/dL
Mean corpuscular volume	90.6	80.0-96.1 fL
Mean corpuscular hemoglobin	30.7	27.3-33.7 pg
Mean corpuscular hemoglobin concentration	339	328-354 g/L
Leukocytes	8.0	4.0-10.0 x 10^9^/L
Neutrophils	83	40.0-80.0%
Lymphocytes	7.9	20.0-40.0%
Monocytes	8.3	2.0-11.7%
Eosinophils	0.3	1.0-6.0%
Basophils	0.5	0.0-2.0%
Platelets	202	150-400 x 10^9^/L
International normalized ratio	1.1	1.0-3.0
Blood chemistry		
Urea	39	13-43 mg/dL
Creatinine	1.02	0.7-1.2 mg/dL
Albumin	3.6	3.5-5.2 g/dL
Glucose	79	70-90 mg/dL
HbA1C	4.9	<5.7%
Electrolytes		
Sodium	135	136-145 mmol/L
Potassium	3.67	3.5-5.1 mmol/L
Chloride	100	98-107 mmol/L
Calcium	13.9	8.6-10.0 mg/dL
Phosphorus	1.1	2.5-4.5 mg/dL
Magnesium	1.5	1.6-2.6 mg/dL
Hormones		
Parathyroid hormone	1,012	15-65 pg/mL
Vitamin D 25-OH-calciferol	25.8	75-250 nmol/L
C-reactive protein	0.1	<0.5 mg/dL
Thyroid-stimulating hormone	0.4	0.27-4.20 mUI/L
Prolactin	12.2	3-15 ng/mL
Insulin-like growth factor 1	121	85-330 ng/mL
Cortisol	9.3	5-25 µg/dL
Follicle-stimulating hormone	2.63	1.4-18.1 U/L
Luteinizing hormone	3.21	1.5-9.3 U/L
Urine		
Protein/creatinine ratio	0.2	<0.2 mg/g
24-hour urinary calcium	429 mg/24h (43,00 mL diuresis)	100-300 mg/24h

**Figure 1:**
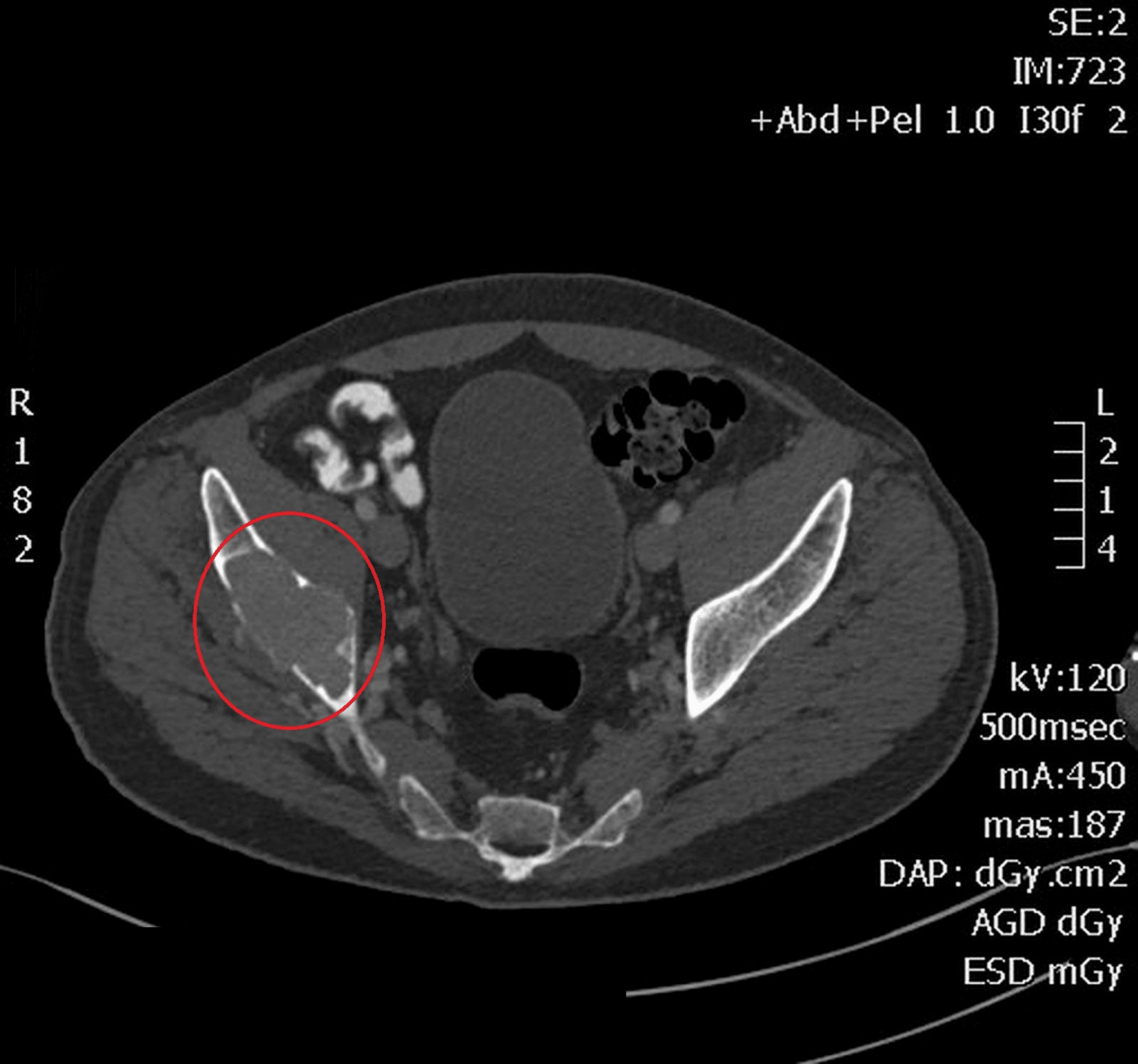
Expansile lytic lesion with well-defined borders in the right iliac bone.

The electrocardiogram showed a shortened QT interval. Computed tomography (CT) of the lumbar spine and pelvis revealed multiple osteolytic lesions, including a large peri-acetabular lesion in the right iliac wing with cortical disruption (Figure 1).

He received intravenous fluids, furosemide, and a single dose of intravenous zoledronic acid for symptomatic hypercalcemia. He was admitted to the Internal Medicine department for further evaluation of moderate-to-severe hypercalcemia with lytic bone lesions.

Investigation

**Figure 2:**
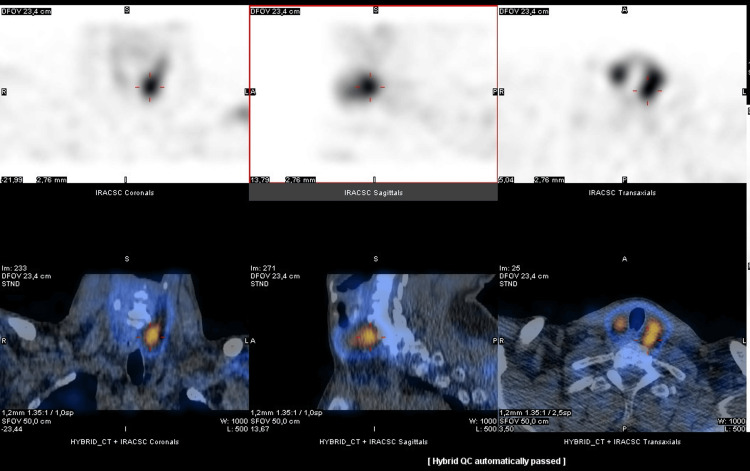
Fused SPECT/CT parathyroid scintigraphy with 99mTc-MIBI showing a focus of increased radiotracer uptake posterior to the inferior pole of the left thyroid lobe, consistent with a hyperfunctioning parathyroid lesion. Coronal, sagittal, and axial views are displayed to illustrate both anatomical localization and functional activity. SPECT/CT, single-photon emission computed tomography/computed tomography; 99mTc-MIBI, technetium-99m sestamibi

To investigate the cause of PHPT with markedly elevated PTH, cervical ultrasound and four-dimensional contrast-enhanced CT were performed. Ultrasound revealed a solid, hypoechoic, well-defined nodule with peripheral vascularization in the right inferior parathyroid region (12 × 12 × 14 mm; 1.06 cm³). A larger left-sided nodule (22 × 16 × 17 mm; 3.14 cm³) showed infiltrative features extending into the adjacent thyroid lobe. Four-dimensional CT confirmed these findings, with a preserved cleavage plane on the right and thyroid infiltration on the left. Single-photon emission computed tomography (SPECT) with technetium-99m sestamibi (99mTc-MIBI) demonstrated hyperfunctioning parathyroid tissue in the left inferior region (Figure 2).

To assess the extent of skeletal involvement and exclude other target organ damage, additional imaging was performed. CT scans of the skull, cervical, thoracic, and lumbar spine, as well as the lower limbs, revealed multiple lytic bone lesions. These were located in the left rib, scapula, clavicle, both iliac bones (including the previously identified right iliac lesion), and the bilateral anterior tibial tuberosities.

Bone mineral density and trabecular bone score (1.261) indicated partially degraded microarchitecture. Densitometry showed low bone density in both trabecular and cortical-rich sites (including the lumbar spine, left femoral neck, and right wrist), consistent with increased fracture risk. Renal ultrasound revealed non-obstructive nephrolithiasis. Biopsy of the right iliac lesion confirmed osteitis fibrosa cystica.

Combined analytical, imaging, and histological findings supported a diagnosis of PHPT due to excessive PTH secretion, presenting with symptomatic hypercalcemia and multiple brown tumors, a presentation that raised early concern for PC.

Treatment

**Figure 3:**
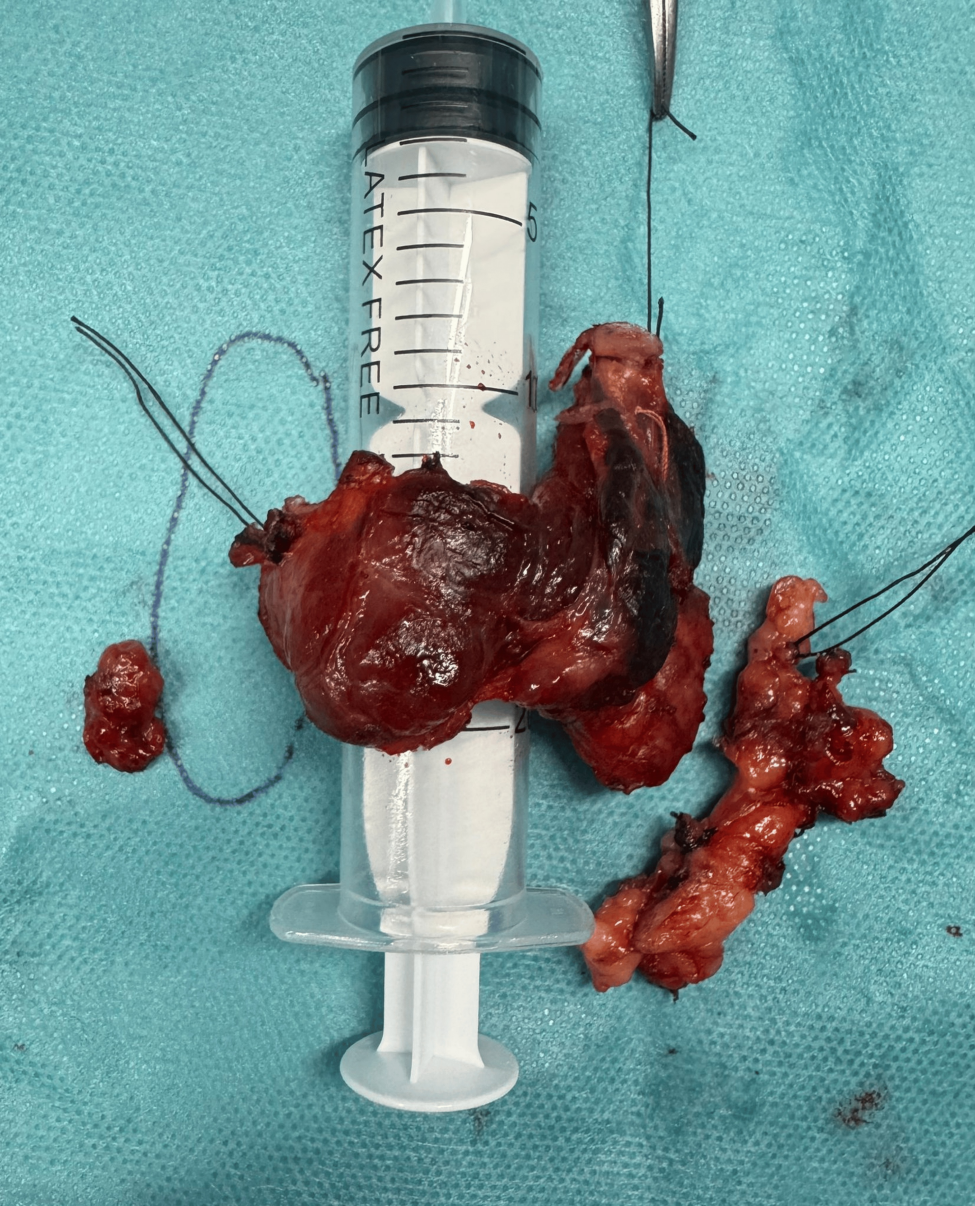
Surgical specimen from en bloc resection, including the left thyroid lobe, isthmus, left recurrent laryngeal nerve, and left inferior parathyroid gland, along with central compartment lymph node dissection. The right inferior parathyroid gland, excised during contralateral exploration, is also shown.

At our institution, cases involving thyroid/PC, suspicious thyroid lesions and hyperparathyroidism are discussed in a multidisciplinary team meeting. This case followed that protocol. Due to two suspicious parathyroid lesions and higher MIBI uptake on the left, initial surgery targeted the left side. An R0 en bloc resection was performed (Figure 3), including the left inferior parathyroid gland, the left thyroid lobe, and the isthmus. Intraoperative recurrent laryngeal nerve (RLN) invasion was noted. Given the patient’s age and the goal of achieving oncologic completeness, the RLN was sectioned. A left central compartment (levels VI and VII) lymphadenectomy was performed with intermittent neuromonitoring.

The intraoperative PTH dropped >50% (from 1,073 to 335 pg/mL at 10 minutes post-excision) but remained relatively stable thereafter (289 pg/mL at 15 and 30 minutes; 315 pg/mL at 1 hour). These findings prompted surgical exploration of the right side, where preoperative imaging had suggested an enlarged right inferior parathyroid gland. Surgical dissection was extended to include the carotid sheath, retroesophageal space, superior mediastinum, thyrothymic ligament, and right thyroid lobe. No ectopic glands were found, but the enlarged right inferior parathyroid gland was excised. Following excision, PTH levels dropped significantly (60 pg/mL at 10 minutes; 47.1 pg/mL at 30 minutes), confirming the gland's contribution to the elevated PTH levels. Additional intraoperative PTH measurements were subsequently recorded: 41.7 pg/mL at 1 hour and 18.8 pg/mL at 4 hours.

The procedure concluded with an end-to-end RLN neurorrhaphy using Prolene® 6/0, covered with dura mater and Tisseel®. A suction drain was placed in the left central compartment.

Outcome

On postoperative day 1, the patient was stable, breathing comfortably on room air, and progressed from a liquid to a light diet. Mild dysphonia was observed, with no paresthesias and a negative Chvostek sign. Laryngoscopy on postoperative day 2 revealed left vocal cord paresis. Speech and swallowing therapy were initiated according to the Voice Unit protocol.

Due to the severity of PHPT and the abrupt postoperative drop in PTH, the patient developed hungry bone syndrome (HBS), with persistent hypocalcemia requiring intravenous calcium and oral calcitriol. Diagnosis was based on serial blood tests showing low calcium and phosphate with normal PTH, consistent with increased bone uptake. HBS manifested within 48 hours post-parathyroidectomy. The patient was discharged on day 20 with calcium carbonate, calcitriol, and cholecalciferol.

**Figure 4:**
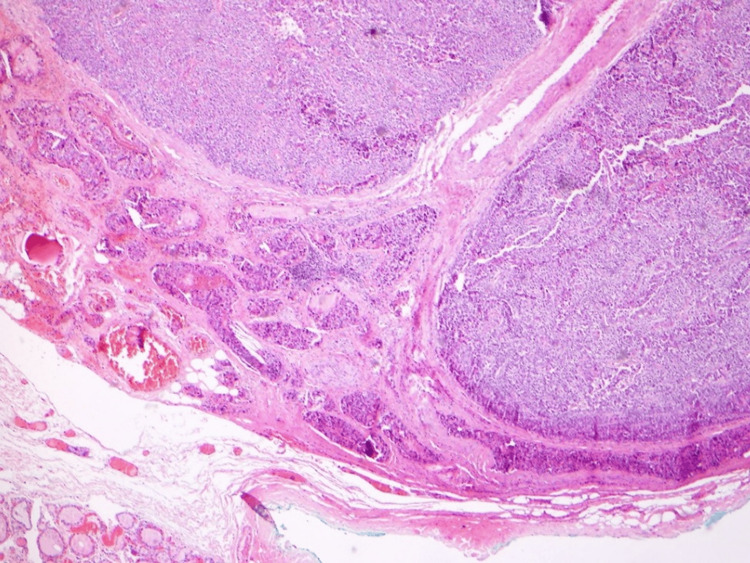
Histopathological image of low-grade parathyroid carcinoma (H&E, 40×). The tumor, originating from the left inferior parathyroid gland, shows fibrous septa, capsular and vascular invasion, and direct extension into adjacent thyroid tissue, supporting the diagnosis of carcinoma (pT2N0R1).

**Figure 5:**
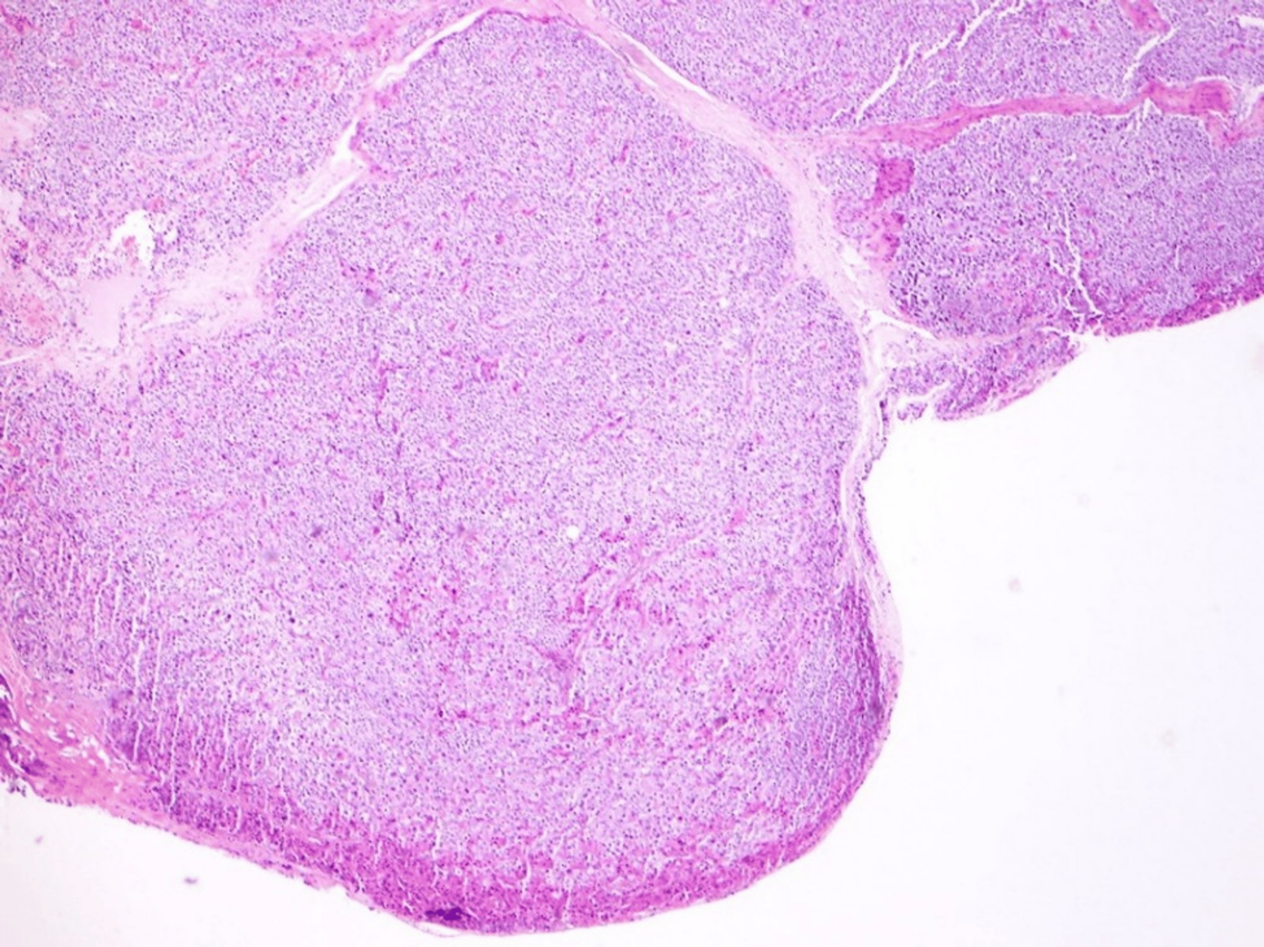
Histopathological image of atypical parathyroid neoplasm (H&E, 40×). The lesion, arising from the right inferior parathyroid gland, lacks definitive features of carcinoma, such as capsular or vascular invasion, and was classified as atypical based on histological criteria and limited resection margins.

Histopathological analysis confirmed a low-grade PC in the left inferior gland (Figure 4), based on the presence of fibrous septa, capsular and vascular invasion, and direct extension into the adjacent thyroid tissue. The tumor was staged as pT2N0R1 according to the American Joint Committee on Cancer (AJCC) Staging Manual, 8th Edition [6,7]. In contrast, the right inferior parathyroid exhibited features of an atypical neoplasm (Figure 5), lacking definitive criteria for carcinoma such as capsular or vascular invasion. The diagnosis was supported by both the initial pathology report and a subsequent slide review, which were consistent in their interpretation. Central lymph nodes showed no pathological alterations.

Genetic testing identified a pathogenic germline mutation in the* CDC73* gene (c.766_767del, p.Val256Lysfs*10), confirming a genetic basis for the disease. Testing was performed using a next-generation sequencing panel targeting hereditary endocrine tumor genes. Variant classification followed American College of Medical Genetics and Genomics/Association for Molecular Pathology (ACMG/AMP) guidelines, based on predicted protein impact, population frequency, and prior clinical evidence [8]. This frameshift mutation results in premature truncation of parafibromin, a tumor suppressor protein whose loss of function is strongly associated with PC, particularly in familial syndromes such as hyperparathyroidism-jaw tumor syndrome (HPT-JT) and familial isolated hyperparathyroidism (FIHP) [9,2]. The patient was referred for genetic counseling and familial risk assessment given the autosomal dominant inheritance pattern and the estimated 50% chance of transmission to offspring [2]. Family members were advised to undergo genetic testing due to the increased risk of developing PC and other *CDC73*-related manifestations [10].

Follow-up

One year after surgery, the patient remains under close follow-up by Internal Medicine, Endocrine Surgery, and Endocrinology. Laboratory results show stable parameters: serum calcium 8.7 mg/dL, phosphate 2.2 mg/dL, and mildly elevated PTH (72.3 pg/mL). Neck ultrasound revealed no abnormalities in the residual thyroid lobe or cervical lymph nodes. Clinically, the patient remains asymptomatic, with no musculoskeletal complaints or signs of recurrence.

## Discussion

PC is a rare but aggressive cause of PHPT, often presenting with severe hypercalcemia and end-organ damage [11,12]. In this case, the diagnosis was supported by markedly elevated calcium and PTH levels, skeletal involvement, and imaging findings consistent with thyroid invasion.

According to the AJCC (8th edition), PC is considered locoregionally advanced at stage T2 or higher, which includes invasion of the thyroid gland [6]. However, due to its rarity, staging remains controversial, and distant metastases remain the most reliable predictor of poor prognosis [7,13].

Surgical management relies on en bloc resection, which remains the only potentially curative approach. The European Society of Endocrine Surgeons recommends ipsilateral hemithyroidectomy and bilateral central compartment lymphadenectomy to achieve R0 resection [7]. In this case, RLN invasion warranted nerve resection and immediate repair, as described in the literature.

Persistent PTH elevation after initial resection led to contralateral exploration, which confirmed a second hyperfunctioning gland. Histological analysis revealed carcinoma on the left and an atypical neoplasm on the right. A *CDC73* mutation confirmed hereditary PC, highlighting the importance of genetic testing for diagnosis, prognosis, and family screening [2,5].

Postoperatively, the patient developed HBS, a known complication in high bone turnover states. HBS results from an abrupt decline in PTH levels, leading to hypocalcemia, hypophosphatemia, and hypomagnesemia [14]. Risk factors include elevated preoperative calcium and PTH levels, large tumor size, osteitis fibrosa cystica, and vitamin D deficiency [1], all of which were present in this case. Although prevention is challenging, preoperative optimization with vitamin D, bisphosphonates, and possibly calcimimetics may help mitigate its severity [15,16]. The use of loop diuretics should be avoided [17].

## Conclusions

This case illustrates the complexity of diagnosing and managing hereditary PC with multiglandular involvement, emphasizing the need for multidisciplinary care, precise surgical planning, and long-term follow-up due to recurrence risk and potential life-threatening hypercalcemia. Given the histopathological findings of the right parathyroid (an atypical neoplasm) with uncertain capsular invasion due to the absence of surrounding tissue, and the presence of a *CDC73* mutation, a more extensive surgical approach, including total thyroidectomy, could have been considered to ensure R0 oncologic status. The remaining thyroid lobe warrants close follow-up, with serial PTH and calcium monitoring to enable early detection of recurrence, as the disease may present aggressively and become fatal if not identified in time.
